# LightSpot^®^-FL-1 Fluorescent Probe: An Innovative Tool for Cancer Drug Resistance Analysis by Direct Detection and Quantification of the P-glycoprotein (P-gp) on Monolayer Culture and Spheroid Triple Negative Breast Cancer Models

**DOI:** 10.3390/cancers13164050

**Published:** 2021-08-11

**Authors:** Antoine Goisnard, Pierre Daumar, Clémence Dubois, Corinne Aubel, Manon Roux, Marie Depresle, Jean Gauthier, Bernard Vidalinc, Frédérique Penault-Llorca, Emmanuelle Mounetou, Mahchid Bamdad

**Affiliations:** 1 Institut Universitaire de Technologie, Université Clermont Auvergne, UMR INSERM-UCA, U1240, Imagerie Moléculaire et Stratégies Théranostiques, 63000 Clermont Ferrand, France; antoine.goisnard@uca.fr (A.G.); pierre.daumar@uca.fr (P.D.); clemence.dubois@uca.fr (C.D.); emmanuelle.mounetou@inserm.fr (E.M.); 2 BIOMARQUEURS Company, 5 Avenue Blaise Pascal, 63178 Aubière, France; manon.roux@uca.fr (M.R.); marie.depresle@uca.fr (M.D.); jean.gauthier@uca.fr (J.G.); bernard.vidalinc@uca.fr (B.V.); 3 Faculté de Médecine, Université Clermont Auvergne, UMR INSERM-UCA, U1240, Imagerie Moléculaire et Stratégies Théranostiques, 63000 Clermont Ferrand, France; corinne.aubel@uca.fr; 4 BIOPASS Company, 5 Avenue Blaise Pascal, 63178 Aubière, France; 5 Centre de Lutte Contre le Cancer Jean Perrin, Université Clermont Auvergne, UMR INSERM-UCA, U1240, Imagerie Moléculaire et Stratégies Théranostiques, 63000 Clermont Ferrand, France; frederique.penault-llorca@clermont.unicancer.fr

**Keywords:** multidrug resistance, P-gp staining, LightSpot^®^-FL-1 cell-permeant fluorescent probe, triple-negative breast cancer

## Abstract

**Simple Summary:**

Tumoral drug resistance is mainly caused by multidrug resistance transporters (MDR), such as the P-gp, which presents high clinical interest. For this reason, the P-gp-mediated drug resistance diagnosis may be very relevant for optimizing anticancer treatment efficacy. However, the lack of effective analytical tools limits this clinical diagnostic approach. Therefore, our group has developed LightSpot^®^-FL-1, a new cell-permeant fluorescent probe able to specifically localize and quantify the P-gp inside unicellular, monolayer, and cellular mass models. The application of this innovative tool was firstly demonstrated in the preclinical field, using five triple-negative breast cancer (TNBC) cell models. The comparison between classical anti-P-gp immunostaining and LightSpot^®^-FL-1 P-gp staining highlighted a strong similarity with P-gp localization and expression level quantification. LightSpot^®^-FL-1 P-gp detection and quantification, using several fluorescence imaging methods, are easy, direct, and cost-effective and are, therefore, very promising for future clinical diagnosis development.

**Abstract:**

P-gp is the most widely studied MDR protein conferring cellular resistance to many standard or targeted therapeutic agents. For this reason, P-gp chemoresistance evaluation, established before or during chemotherapy, can be very relevant in order to optimize the efficacy of treatments, particularly for aggressive tumoral subtypes such as triple-negative breast cancer (TNBC). In this context, our team developed an innovative cell-permeant fluorescent probe called the LightSpot^®^-FL-1, which is able to specifically localize and quantify the P-gp in cells or cell masses, as evidenced on different TNBC cell models. First, flow cytometry analysis showed LightSpot^®^-FL-1 cell penetration and persistence in time, in TNBC cells. Then, LightSpot^®^-FL-1 staining was compared to anti-P-gp immunostaining by fluorescence microscopy on five TNBC cell lines. Results showed a clear similarity of P-gp localization and expression level, confirmed by Pearson’s and Mander’s colocalization coefficients with 92.1% and 100.0%, and a strong correlation coefficient of R^2^ = 0.99. In addition, the LightSpot^®^-FL-1 staining allowed the quantification of a P-gp induction (33% expression increase) following a 6-hour spheroid model exposure to the anti-PARP Olaparib. Thus, the new LightSpot^®^-FL-1 cell-permeant probe, targeting P-gp, appears to be an effective tool for drug resistance evaluation in preclinical models and shows promising possibilities for future use in clinical diagnosis.

## 1. Introduction

The permeability-glycoprotein (P-gp) was discovered about forty years ago, initially as a membrane transporter at the origin of chemoresistance in cancer [1]. In reality, P-gp is part of a wide class of “ATP-binding cassette” (ABC) superfamily transporter proteins, called “multidrug resistance” (MDR). MDR proteins, and the P-gp in particular, were highly conserved during the evolutionary process and are ubiquitous. In fact, they are naturally expressed in a wide variety of living organisms from prokaryotes to eukaryotes, such as bacteria, protozoa, fish, *Drosophila* and mammals [2,3,4,5,6]. In humans, MDR proteins are commonly studied because of their involvement in tumoral multidrug resistance mechanisms [7,8]. Indeed, MDR proteins act as xenobiotic transmembrane efflux pumps, recognizing a large panel of substrates with different structures and properties, such as various pollutants and drugs [9,10,11]. These pumps establish a veritable cellular defense network, exhibiting a protective role against external aggressions. At present, MDR transporters are considered to be an essential part of the innate cellular defense system, also called the “chemoimmunity” network, that presents various characteristics reminiscent of classical immunology [12]. This system is particularly complex because a single MDR transporter can recognize a wide variety of substrates, and one anticancer agent can also be the target of several transporters. Thus, MDR proteins have been identified as one of the main causes of tumoral cell cross-resistance against anticancer drugs. Indeed, many conventional chemotherapeutic agents, such as anthracyclines, taxanes or vinca-alkaloids, and/or targeted therapeutics such as tyrosine kinase inhibitors or poly-ADP ribose polymerase inhibitors (anti-PARPs) are expelled from cells by MDR transporters [13,14,15], and many tumoral types overexpress several MDR transporters. The main identified MDR proteins presenting a clinical involvement are (i) the P-gp encoded by *ABCB1*, (ii) the MDR-associated proteins (MRP) encoded by *ABCC* genes, such as MRP1 (*ABCC1*) or MRP7 (*ABCC10*), and (iii) the breast cancer resistance protein (BCRP) encoded by the *ABCG2* gene [8,16].

Numerous biological mechanisms can lead to the development of chemoresistance, among which MDR-mediated drug efflux is the most frequently described [17]. Likewise, high expression of MDR proteins is often associated with aggressive subtypes and low disease-free survival rates in breast tumors [18]. Thereby, their clinical relevance resides in their predictive value on tumoral drug resistance, which is directly linked to the prognosis of the disease [19,20]. Thus, the importance of considering the P-gp expression level in tumors to optimize patients’ treatment orientation was highlighted in several studies [19,21,22]. However, the P-gp expression level is not frequently evaluated in clinical practice, which can in part be attributed to one specific issue, namely the lack of relevant analytical tools [8].

Triple-negative breast cancer (TNBC) is a breast cancer subtype that is immunohistochemically negative for the estrogen receptor (ER) and for the progesterone receptor (PR), and is also associated with the absence of HER2 protein overexpression and/or *HER2* gene amplification [23]. This heterogeneous and aggressive disease accounts for 15 to 20% of breast cancers and is associated with poor prognosis and drug resistance [17,24,25]. Despite colossal efforts made regarding TNBC treatments, only 20% of patients present a pathologically complete response after treatment, while others are most likely to present an early recurrence or develop a metastatic disease [26]. As a matter of fact, TNBC tumors are, at first, particularly sensitive to chemotherapy, but a high proportion becomes resistant during treatment and presents difficulties for achieving a complete pathological response. This phenomenon is called the “triple-negative paradox” and explains why TNBC represents a relevant study model for cancer-cell drug resistance [17,27].

In the last few years, the works of our research group were focused on optimizing TNBC treatment, in correlation with resistance mechanisms mediated by MDR proteins. Indeed, we highlighted the relay overexpression of BCRP and P-gp MDR proteins on SUM1315 monolayer TNBC cell cultures, after exposure to the PARP inhibitor Olaparib [28]. Our group has developed various in vitro preclinical tools, e.g., TNBC cancer spheroids mimicking avascular small-size micro-tumors [29,30,31,32], notably for realizing high-throughput drug screening or modeling the distribution of small molecules of interest inside these cell masses [33]. In parallel, through pharmaco-modulation approaches, we designed bio-active fluorescent compounds such as LightSpot^®^ fluorescent tracers targeting P-gp [34].

In these present works, we report on the validation of the LightSpot^®^-FL-1 tracer as an innovative fluorescent probe for selective and sensitive detection, localization, and quantification of the P-gp in cells or cell masses. The proof of concept was established on several unicellular, monolayer or spheroid TNBC models and a microscopy-based methodology was performed to explore native or induced P-gp expression on those models.

## 2. Experimental Section

### 2.1. LightSpot^®^-FL-1 Chemical Synthesis Achievement

Commercially available reagents and solvents were used without further purification for the synthesis and characterization of LightSpot^®^-FL-1, as previously described [34]. Briefly, LightSpot^®^-FL-1 was obtained by coupling an amine-bearing P-gp inhibitor derivative with the commercial amine-reactive *N*-hydroxysuccinimidyl ester of the BODIPY-FL fluorophore (CAS 146616-66-2, Interchim, Montluçon, France). After the coupling reaction proceeded, a preparative High-Performance Liquid Chromatography (HPLC) purification system (Shimadzu Inc., Kyoto, Japan) was performed to afford LightSpot^®^-FL-1 in good yield (>70%). LightSpot^®^-FL-1 was isolated at > 96% purity based on HPLC-UV-DAD analysis, and fully characterized by ^1^H NMR, ^13^C NMR, 2-dimensional COSY and HSQC NMR, and high-resolution mass spectrometry. Excitation and emission maxima were determined from LightSpot^®^-FL-1 solutions in ethanol at 505 and 510 nm, respectively. Pure dry aliquots of LightSpot^®^-FL-1 were stored at −20 °C and were used to prepare 1.0 mM stock solutions in DMSO for biological experiments.

### 2.2. Monolayer Cell Culture and Spheroid Formation

Triple-negative breast cancer SUM1315 (MO2, ASTERAND), MDA-MB-231 (ATCC^®^, HTB-26™), HCC1937 (ATCC^®^ CRL-2336™), SW527 (ATCC^®^ CRL-7940™) and DU4475 (ATCC^®^, HTB-123™) cell lines were stored in the Biological Resource Center of the Jean Perrin Comprehensive Cancer Center, identified under No. BB-0033-00075 (Clermont-Ferrand, France). Each cell line was checked for the absence of mycoplasma contamination (Mycoplasmacheck^®^ test, Eurofins Genomics, Luxembourg, Luxembourg) and maintained in their recommended cell culture media at 37 °C under 5% CO_2_ in a humidified incubator. For monolayer cell culture, SUM1315 cells were cultured in Ham’s F-12 medium (Gibco, Dublin, Ireland), supplemented with 5% FCS (fetal calf serum, Eurobio Scientific, Paris, France), 10 mM HEPES buffer (Sigma, Darmstadt, Germany), 4 µg/mL insulin (Novo Nordisk, Bagsværd, Denmark), 10 ng/mL epidermal growth factor (EGF, Sigma, Darmstadt, Germany) and 20 µg/mL gentamicin (Panpharma, Paris, France). MDA-MB-231, HCC1937 and DU4475 cells were cultured in RPMI 1640 medium (Gibco, Dublin, Ireland) supplemented with 10% decomplemented fetal calf serum and 20 µg/mL gentamicin. SW527 cells were cultures in DMEM high-glucose medium (Gibco, Dublin, Ireland) supplemented with 10% decomplemented fetal calf serum and 20 µg/mL gentamycin.

Spheroids were formed with the SUM1315 TNBC cell line, according to our previous works [29]. Briefly, cells in suspension were seeded in ultra-low-attachment round-bottom microplates in a prepared culture medium (Corning, New York, NY, USA, catalog #4520) at a concentration of 1000 cells/well. After 24 h, Geltrex^®^ reduced growth factor basement membrane matrix (Gibco, Dublin, Ireland), diluted with cold medium, was dispensed in each well for a final concentration of 2%. Microplates were then horizontally shacked for 20 min at 200 rpm, before being replaced in the incubator.

### 2.3. Lightspot^®^-Fl-1 Cellular Uptake and Outtake Analysis by Flow Cytometry

Suspension DU4475 and monolayer SUM1315 cell cultures were used, according to the culture conditions described above. For SUM1315, cell detachment was achieved by trypsinization. Then, cellular suspensions containing 800,000 cells/mL were prepared in a culture medium for both cell lines. Cells maintained at 37 °C in the culture medium were then incubated with 1 µM fluorescent LightSpot^®^-FL-1 compound, dissolved in DMSO and diluted in the culture medium. The final DMSO concentration in all cell culture conditions was at 0.1%. Fluorescence intensity in cells was measured after 5, 15, 30, 45 and 60 min using a flow cytometer LSRII (Becton Dickinson, Franklin Lakes, NJ, USA) with a blue excitation laser (488 nm, 20 mW) and FITC filter (530/30 nm). Cells were then centrifuged (125× *g* for 5 min) in order to be washed. For this process, the cell pellet was resuspended in the cell culture medium at 37 °C. Fluorescence intensity was then monitored after 5, 15, 60 and 120 min, following the washing step.

### 2.4. LightSpot^®^-FL-1 Fluorescent Staining on Monolayer and Suspension Cell Cultures

For the adherent SUM1315 cell line, cells were seeded in IbiTreat 8-well µ-Slides (Ibidi^®^, Gräfelfing, Germany) at a concentration of 50,000 cells per well. Slides were maintained at 37 °C under 5% CO_2_ in a humidified incubator until the cell carpet reached 60% of confluence. For the suspension DU4475 cell line, cells were directly harvested from the culture flask. In both cases, cells were either fixed with a 4% paraformaldehyde (PFA) solution or were maintained unfixed in culture conditions. For both fixed and unfixed cells, all subsequent experimental and imaging steps were achieved in the same experimental conditions. Cells were then incubated in a 1 µM LightSpot^®^-FL-1 D-PBS solution for one hour. Three successive washes of 20 min were then conducted in D-PBS to remove unspecific cell staining. Nuclear staining was carried out using 1 µg/mL bisbenzimide Hoechst33258 solution (Sigma Aldrich, Darmstadt, Germany, catalog #14530). Cells were finally imaged with a Cytation™3 MV (BioTek^®^, Winooski, VT, USA) fluorescent microscopy module (M = 40, X-fluorescence filters = GFP, LED = 10, time exposure = 200 ms, gain = 10). The cells’ mean fluorescence intensity was calculated with Gen5 3.08 software (BioTek^®^, cellular analysis, threshold = 4000, minimal size = 5 µm, maximal size = 100 µm).

### 2.5. LightSpot^®^-FL-1 and Anti-P-gp Co-staining Analysis on Monolayer and Suspension Cell Cultures

LightSpot^®^-FL-1 staining was conducted as previously described for SUM1315, MDA-MB-231, HCC1937, SW527 and DU4475 cell lines, before completing the anti-P-gp immunostaining. For this, cells were incubated with clone F4 anti-P-gp monoclonal mouse antibody (Invitrogen, Carlsbad, CA, USA, catalog # MA5-13854, diluted at 1/75) for one hour. Three successive washes were then carried out in D-PBS before incubating cells with secondary goat anti-mouse Alexa Fluor™ 647 nm antibody (Invitrogen, Carlsbad, CA, USA, catalog #A21236 diluted at 1/800). Cells were finally imaged with a Cytation™3 MV (BioTek^®^, Winooski, VT, USA) fluorescent microscopy module (M = 40, X-fluorescence filters = GFP, LED = 10, time exposure = 200 ms, gain = 10/Cy 5, LED = 10, time exposure = 1840 ms, gain = 16). The GFP and Cy5 cells’ mean fluorescence intensity were calculated with Gen5 3.08 software (BioTek^®^, cellular analysis, primary mask set on GFP filter, threshold = 4000, minimal size = 5 µm, maximal size = 100 µm). For DU4475 cells presenting the strongest signals among tested cell lines, a colocalization study was made using the Coloc 2 plugin of ImageJ software (NIH, Bethesda, MD, USA). This allowed us to calculate Pearson’s correlation and Mander’s overlap coefficients between the GFP- and Cy5-acquired images.

### 2.6. LightSpot^®^-FL-1 Fluorescent Staining on SUM1315 Spheroids

After 3 days of culture, the spheroids were harvested and fixed for 12 h in 4% PFA solution. They were then incubated for 90 min in 1 µM LightSpot^®^-FL-1 solution. Then, spheroids were imaged with the Cytation™3 MV (BioTek^®^, Winooski, VT, USA) fluorescent microscopy module (M = 4, X-fluorescence filters = GFP, LED = 10, time exposure = 150 ms, gain = 4) 30, 60, 90 and 120 min after the end of LightSpot^®^-FL-1 incubation. Between each observation, the spheroids were washed in D-PBS. The spheroid global fluorescent intensity was measured with Gen5 3.08 software (BioTek^®^, cellular analysis on GFP filter, threshold = 5000, minimal size = 100 µm, maximal size = 500 µm).

### 2.7. P-gp Expression Level Measurement in SUM1315 Monolayer Cell Line and in Spheroid Models after Olaparib Treatment

Olaparib (Carbosynth, Compton, catalog #FO33122) was solubilized in DMSO to prepare a 50 mM stock solution. Dilutions were prepared in Ham’s F-12 medium for final treatment concentrations of 0.5, 5 or 50 µM. The final DMSO concentration remained constant at 0.1% in all tested conditions.

For the SUM1315 monolayer culture, cells were seeded in IbiTreat 8-well µ-Slides (Ibidi^®^, Gräfelfing, Germany) at a concentration of 50,000 cells per well and maintained in a humid incubator until 60% of confluency. Cells were exposed for 3 h to 0.5, 5 or 50 µM of Olaparib and then fixed for 10 min with PFA at 4%. This drug was solubilized in DMSO, and the final DMSO concentration was maintained at 0.1% in both treated and untreated conditions. For each Olaparib concentration, the PFA-fixed cells were then stained with 1 µM LightSpot^®^-FL-1 or with anti-P-gp immunostaining, as previously described above. For the immunostaining, goat anti-mouse primary antibody (Invitrogen, Carlsbad, CA, USA, catalog #MA5–13854, diluted at 1/75) was coupled with Alexa Fluor™ 568 mn (Invitrogen, Carlsbad, CA, USA). Images were acquired with a Cytation™3 MV (BioTek^®^, Winooski, VT, USA) fluorescent microscopy module (M = 40, X-fluorescence filters = GFP, LED = 10, time exposure = 200 ms, gain = 10/Texas Red, LED = 10, time exposure = 2200 ms, gain = 16). The fluorescence intensities of GFP and Texas Red signals were calculated using the Gen5 3.08 software (BioTek^®^, cellular analysis, threshold = 4000 for GFP signal and 3100 for Texas Red signal, minimal size = 50 µm, maximal size = 100 µm).

SUM1315 spheroids were exposed for 3 or 6 h with 0.5, 5 or 50 µM of Olaparib. In parallel, 0.1% DMSO controls were also performed. After treatment, spheroids were fixed in 4%PFA solution and incubated for 90 min with LightSpot^®^-FL-1 diluted at 1 µM. They were then washed for 90 min in D-PBS and were observed with a Cytation™3 MV (BioTek^®^, Winooski, VT, USA) fluorescent microscopy module (M = 4, X-fluorescence filter = GFP, LED = 10, time exposure = 150 ms, Gain = 4). Spheroid global fluorescent intensity was measured using Gen5 3.08 software (BioTek^®^, cellular analysis on GFP filter, threshold = 5000, minimal size = 100 µm, maximal size = 500 µm).

### 2.8. Statistical Analysis

Results were presented as mean ± standard deviation. All experiments were performed independently on at least three separate occasions. Unpaired 2-sided Student’s *t*-test or ANOVA and Tukey’s multiple comparison test were used to evaluate statistical significance. Results were considered statistically different when *p* < 0.05 (*). The strongest differences were noted as follows: *p* < 0.01 (**), *p* < 0.001 (***), *p* < 0.0001 (****) and *p* < 0.00001 (*****). Non-significant results were noted as “ns”.

## 3. Results

### 3.1. Synthesis and Characterization of the LightSpot^®^-FL-1 Probe

**Figure 1:**
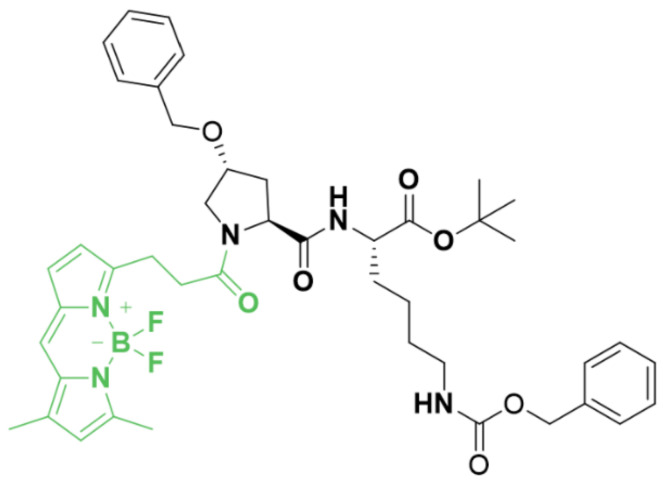
Chemical structure of LightSpot^®^-FL-1: a peptidic P-gp ligand (black) conjugated to the BODIPY-FL fluorophore (green).

The structure of LightSpot^®^-FL-1 presents a peptidic moiety that acts as a P-gp ligand, conjugated via an amide bond to the cell-permeant, bright, and photostable BODIPY-FL fluorophore (Figure 1). The amide bond was formed by the reaction of an amine-bearing peptidic P-gp ligand with BODIPY-FL succinimidyl ester. Subsequent HPLC purification led to LightSpot^®^-FL-1, with a good yield (>70%) and excellent purity (>96%). After full characterization, the excitation and emission maxima were determined from LightSpot^®^-FL-1 solutions in ethanol at 505 and 510 nm, respectively.

### 3.2. LightSpot^®^-FL-1 Fluorescent Probe Selectively Targets P-gp in the DU4475 and SUM1315 TNBC Cell Line

#### 3.2.1. LightSpot^®^-FL-1 Kinetic Uptake by Flow Cytometry

To assess the impact of the BODIPY-FL conjugation on the ligand moiety spatiotemporal cellular distribution, the ability of LightSpot^®^-FL-1 to penetrate and subsist in cells was evaluated by flow cytometry.

**Figure 2:**
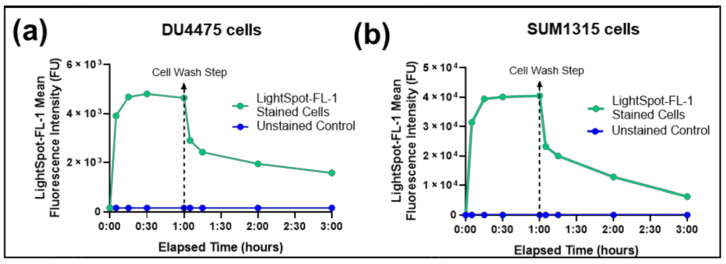
LightSpot^®^-FL-1 kinetic uptake in DU4475 and SUM1315 TNBC cell lines. DU4475 (**a**) and SUM1315 (**b**) TNBC cells were exposed to a 1 µM LightSpot^®^-FL-1 fluorescent probe and the cellular fluorescence intensity (fluorescence units: FU) was measured at different times by flow cytometry analysis. After 1 h of exposure, DU4475 (**a**) and SUM1315 (**b**) cells were washed with culture medium (cell wash step) and the cellular fluorescence intensity was again measured at different times by flow cytometry, 2 h after the cell-wash step. During the entire experiment time, unstained cells were analyzed in parallel as control values.

For this, two TNBC cell line models were used, the suspension DU4475 and the monolayer SUM1315 cell cultures in which the P-gp is naturally overexpressed [28,35]. Both cell lines were exposed to 1 µM LightSpot^®^-FL-1 for 1 h. LightSpot^®^-FL-1 intracellular fluorescence intensity was measured over time, during cell exposure, and after washes to remove the unbound LightSpot^®^-F-L1 (Figure 2a,b). In both cell lines, a rapid LightSpot^®^-FL-1 uptake was detected in the first 15 min, with a mean fluorescence intensity from 127 FU to 5.0 × 10^3^ FU, and from 8 FU to 39.5 × 10^3^ FU for DU4475 and SUM1315 cells, respectively. Then, the intracellular LightSpot^®^-FL-1 rate was stabilized in both cells, with 4.8 × 10^3^ FU for DU4475 cells (Figure 2a) and 46.4 × 10^3^ FU for SUM1315 after 60 min (Figure 2b). After LightSpot^®^-FL-1 rate stabilization, cells were washed to eliminate unbound LightSpot^®^-FL-1. After this step, intracellular LightSpot^®^-FL-1 fluorescence was again measured in each cell line, corresponding to the intracellular LightSpot^®^-FL-1 specifically bound on its P-gp target. Thereafter, a clear decrease of LightSpot^®^-FL-1 fluorescence intensity was detected in both cell lines with 2.9 × 10^3^ FU for DU4475 cells and 23.2 × 10^3^ FU for SUM1315 cells, 5 min after the wash step. The LightSpot^®^-FL-1 maintenance in the cells was then followed in each cell line. In DU4475 cells, the linked LightSpot^®^-FL-1 rate remained relatively stable over time, with 1.9 × 10^3^ FU after 1 h and 1.6 × 10^3^ FU after 2 h (Figure 2a). However, for SUM1315 cells, the intracellular LightSpot^®^-FL-1 rate continued to decrease to 12.9 × 10^3^ FU after 1 h, and to 6.3 × 10^3^ FU after 2 h (Figure 2b). These results showed that the intracellular fluoroprobe LightSpot^®^-FL-1 was maintained in both cell lines over time.

##### Lightspot^®^-FL-1 Staining Intensity Comparison between Living or Fixed Cells by Fluorescence Imaging

**Figure 3:**
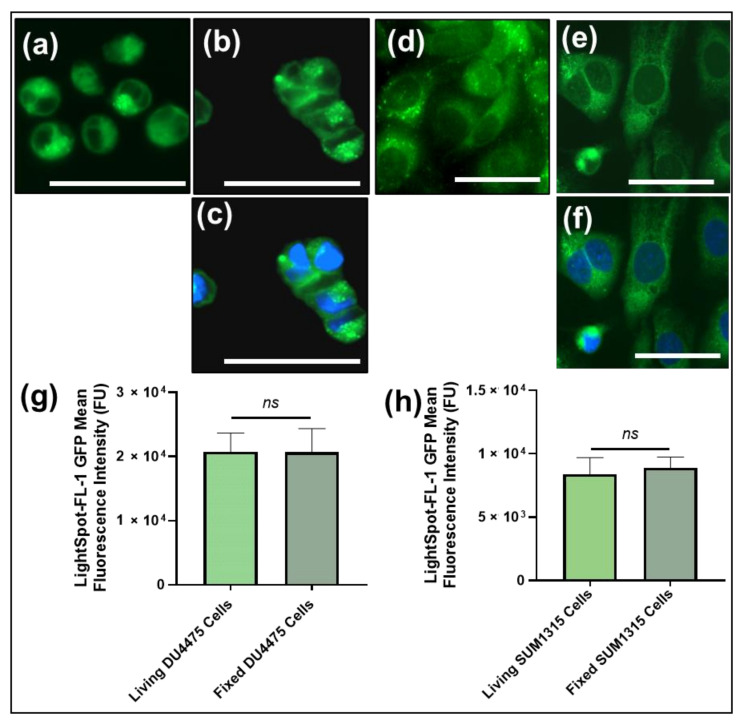
LightSpot^®^-FL-1 staining comparison between living and fixed DU4475 and SUM1315 TNBC cell lines. Living (**a**) or fixed (**b**) DU4475 cells and living (**d**) or fixed (**e**) SUM1315 cells were stained with the LightSpot^®^-FL-1 probe at 1 µM (green signal). In parallel, a nuclear counterstaining was also carried out using Hoechst33258 for fixed DU4475 (**c**) and SUM1315 (**f**) cell conditions (merged pictures presenting LightSpot^®^-FL-1 signal in green and nuclear staining in blue). All pictures were acquired with the Cytation™3 MV (BioTek^®^, M = 4 X, scale bar = 50 µm). For each experimental condition, LightSpot^®^-FL-1 global cell fluorescent intensity was measured with Gen5 software (BioTek^®^) for DU4475 (**g**) and SUM1315 (**h**) cells. Significances between living and fixed conditions were indicated as ns (not significant; *p* > 0.05).

The LightSpot^®^-FL-1 staining efficiency was then analyzed and compared between living and fixed cells. For these experiments, living cells or PFA-fixed cells were incubated with 1 µM LightSpot^®^-FL-1 solution for 1 h before being washed. In the acquired images, DU4475 (Figure 3a–c) and SUM1315 (Figure 3d–f) cells presented the same LightSpot^®^-FL-1 green fluorescent staining profiles between living or fixed conditions. Moreover, no similarities with blue Hoechst33258 staining were observed (Figure 3c,f) indicating an absence of LightSpot^®^-FL-1 compound accumulation in the cell nuclei (Figure 3e,f). LightSpot^®^-FL-1 staining intensity was then quantified and was 20.8 ± 2.9 × 10^3^ FU and of 20.7 ± 3.7 × 10^3^ FU for living and fixed DU4475 cells, respectively (*p* = 0.94; Figure 3g). For the SUM1315 cell line, the staining intensity was 8.4 ± 1.3 × 10^3^ FU and 8.9 ± 0.8 × 10^3^ FU for living and fixed cells, respectively (*p* = 0.17; Figure 3g). These results showed that the intensity of the LightSpot^®^-FL-1 staining was similar on living or fixed cells with similar staining parameters, in terms of distribution and intensity. Therefore, for the following experiments, only fixed-cell models were used.

##### P-gp Co-Staining with LightSpot-FL-1 and Immunostaining

**Figure 4:**
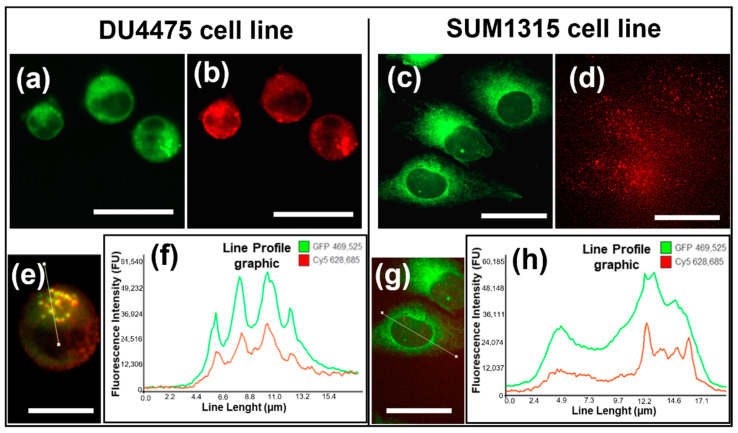
P-gp targeting comparison between LightSpot^®^-FL-1 staining and anti-P-gp immunostaining on DU4475 and SUM1315 TNBC cell lines. Co-staining was carried out, combining LightSpot^®^-FL-1 with anti-P-gp immunostaining. Pictures of LightSpot^®^-FL-1 labeling (green GFP signal) are presented for DU4475 (**a**) and SUM1315 cells (**c**). Similarly, immunostaining pictures (red Cy5 signal) are presented for DU4475 (**b**) and SUM1315 cells (**d**). Merged pictures are presented for DU4475 (**e**) and SUM1315 (**g**) cells. A line profile was drawn on merged pictures, superimposing both LightSpot^®^-FL-1 and anti-Pgp immunostaining signals. The fluorescence intensity spectra across the line profile are shown with the line profile graphic on DU4475 cells (**f**) and SUM135 cells (**h**). All pictures were acquired with the Cytation™3 MV (BioTek^®^, M = 4 X, scale bar = 25 µm).

For the analysis of P-gp targeting by the LightSpot^®^-FL-1 fluorescent probe, the distribution of this intracellular tracer (green signal) was compared to specific immunostaining, combining anti-P-gp mouse antibody (F4 clone) and a goat anti-mouse secondary antibody coupled to Alexa Fluor™ 647 (red signal). For this, co-staining was carried out on both DU4475 and SUM1315 BLTN models. In the unicellular DU4475 cell line (Figure 4a,b,e), a clear similarity of localization was detected between the LightSpot^®^-FL-1 probe labeling (Figure 4a) and the anti-P-gp immunostaining (Figure 4b). On SUM1315, similar staining profiles were obtained (Figure 4c,d,f). These staining similarities were highlighted by line profiles drawn on the DU4475 and SUM1315 merged picture (Figure 4e,f,g,h). Moreover, the colocalization of LightSpot^®^-FL-1 and anti-P-gp staining on DU4475 was also confirmed by Pearson’s correlation coefficient analysis, reaching 92.1%, and by Mander’s overlap coefficient, reaching 100.0%, attesting to similar signal distribution.

### 3.3. LightSpot^®^-FL-1 Allows Sensitive Detection and Quantification of Basal P-gp Expression Level in Various TNBC Cell Lines

The performance of the cell-permeant fluorescent probe LightSpot^®^-FL-1 was then checked by the quantification of P-gp expression level on five various TNBC cell lines, DU4475, SW527, HCC1937, MDA-MB-231 and SUM1315, in comparison with the anti-P-gp immunostaining reference method (Figure 4).

**Figure 5:**
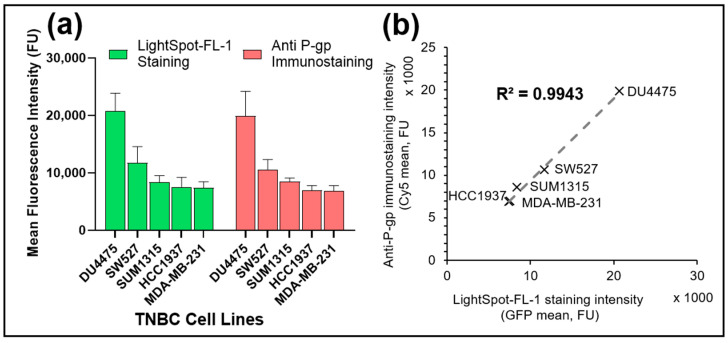
P-gp level quantification comparison after co-staining with LightSpot^®^-FL-1 staining and anti-P-gp immunostaining on several triple-negative breast cancer cell lines. Co-staining was carried out by combining LightSpot^®^-FL-1 staining with anti-P-gp immunostaining on five different TNBC cell lines i.e., DU4475, SW527, HCC1937, MDA-MB-231 and SUM1315. For each cell line, (**a**) both LightSpot-FL staining (green bars) and anti-P-gp immunostaining (red bars) fluorescence intensities were measured using the Gen5 software (BioTek^®^). Correlation between both stainings was evaluated (**b**) with the correlation line and the R^2^ coefficient of determination.

In all TNBC studied models, P-gp signal quantification was proportional between LightSpot^®^-FL-1 labeling and anti-P-gp labeling (Figure 5a). Indeed, the fluorescence intensities of LightSpot^®^-FL-1 labeling were 20.7 ± 3.1 × 10^3^ FU, 11.7 ± 2.9 × 10^3^ FU, 7.6 ± 1.7 × 10^3^ FU, 8.4 ± 1.1 × 10^3^ FU, 7.4 ± 1.1 × 10^3^ FU for DU4475, SW527, HCC1937, MDA-MB-231 and SUM1315 cell lines, respectively (Figure 5a). Likewise, anti-P-gp immunofluorescence intensities were 19.9 ± 4.2 × 10^3^ FU, 10.6 ± 1.8 × 10^3^ FU, 8.5 ± 0.6 × 10^3^ FU, 6.9 ± 0.8 × 10^3^ FU, and 6.9 ± 0.9 × 10^3^ FU for DU4475, SW527, HCC1937, MDA-MB-231 and SUM1315 cell lines, respectively (Figure 5a). Moreover, the fluorescence signal intensities correlation was studied for all cell lines, giving an R^2^ determination coefficient of 0.9943, attesting to a strong correspondence between the intensities of the LightSpot^®^-FL-1 tracer and anti-P-gp signals (Figure 5b). These results showed a strong intensity correlation between the LightSpot^®^-FL-1 staining and the anti-P-gp immunostaining, clearly highlighting the LightSpot^®^-FL-1 probe performance in terms of detection sensitivity for P-gp expression levels in all TNBC models studied.

### 3.4. LightSpot^®^-FL-1 Detects and Quantifies Olaparib-induced P-gp expression Level on SUM1315 Monolayer Cultures

**Figure 6:**
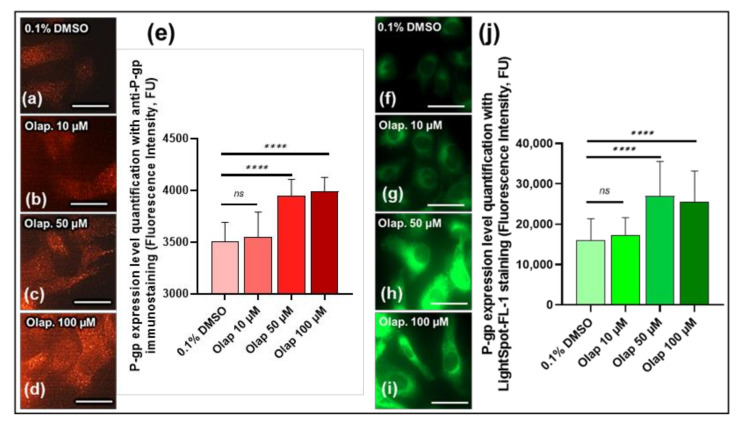
Comparison between anti-P-gp immunostaining and LightSpot^®^-FL-1 fluorescent probe staining for P-gp level expression measure on the SUM1315 cell line in monolayer culture condition after increased Olaparib concentration exposure. SUM1315 cells were treated with 10, 50 or 100 µM Olaparib for 3 h. Cells were then fixed and immunostained, combining anti-P-gp mouse antibody (F4 clone) and a goat anti-mouse secondary antibody, coupled to Alexa Fluor™ 568 (red signal) for each dose (**a**–**d**), and the signal was quantified using Gen5 software (BioTek^®^) (**e**). LightSpot^®^-FL-1 staining (green signal) was also utilized for the same experimental conditions (**f**–**i**), and quantified using Gen5 software (**j**). All pictures were acquired with the Cytation™3 MV (BioTek^®^, M = 4 X, scale bar = 50 µm). Significances between conditions were indicated as ns (not significant; *p* > 0.05), and **** *p* < 0.0001.

Thereafter, the sensitivity of LightSpot^®^-FL-1 fluorescent staining for the quantification of the P-gp expression level was checked, in comparison with anti-P-gp immunostaining on SUM1315 cell lines. For this study, the SUM1315 cells cultured in monolayer were treated for 3 h with increasing concentrations of Olaparib (10, 50 or 100 µM), a PARP inhibitor used for TNBC treatment. For these experiments, Olaparib was dissolved in DMSO. The final concentration of this solvent was always 0.1% in all cell-culture conditions (Figure 6). The fluorescence intensities were normalized to DMSO control values as a basal P-gp expression level. The results showed that, with anti-P-gp immunostaining, the basal P-gp expression level was 3.5 ± 0.2 × 10^3^ FU for the control 0.1% DMSO cells (Figure 6a,e). After 10 µM Olaparib treatment, the P-gp expression remained similar to control, with 3.6 ± 0.2 × 10^3^ FU (Figure 6b,e). In contrast, with 50 and 100 µM Olaparib, a significant increase in P-gp expression level was detected, with 3.9 ± 0.2 × 10^3^ FU and 3.9 ± 0.1 × 10^3^ FU, respectively (*p* = 10^−15^ & 10^−15^, Figure 6c–e), compared to 0.1% DMSO control cells. Very interestingly, a similar P-gp expression level evolution was detected with LightSpot^®^-FL-1 staining (green signal) in the same experimental conditions (Figure 6f–i). Indeed, 10 µM Olaparib-treated cells presented a similar P-gp expression level, with 17.4 ± 4.3 × 10^3^ FU compared to the 0.1% DMSO control presenting 16.1 ± 5.3 × 10^3^ FU (*p* = 0.77, Figure 6f,g,j). In contrast, after 50 and 100 µM Olaparib cell treatment, significantly increased P-gp expression levels were measured with 27.0 ± 8.6 × 10^3^ FU and 25.5 ± 7.7 × 10^3^ FU, respectively (*p* = 10^−14^ & 10^−11^, Figure 6h–j), compared to 0.1 % DMSO controls. These results clearly highlighted the fact that the LightSpot^®^-FL-1 fluorescent probe presented the same P-gp detection and quantification level efficiency as anti-P-gp immunostaining.

### 3.5. The Cell-permeant LightSpot^®^-FL-1 Detects and Quantifies Basal and Induced P-gp Expression in SUM1315 Spheroid Cell Mass Models

**Figure 7:**
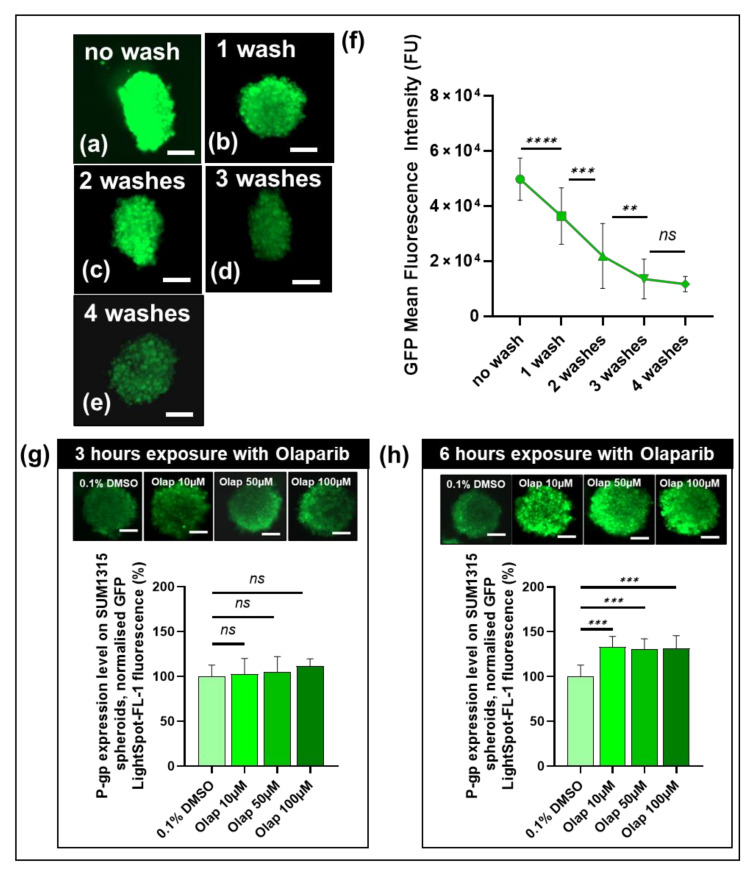
Development of LightSpot^®^-FL-1 staining on SUM1315 spheroids. SUM1315 spheroids (about 200 µm diameter) were incubated with LightSpot^®^-FL-1 at 1 µM and washed several times in D-PBS. Before and between each wash of 30 min, pictures of spheroids were made (**a**) for no wash, (**b**) for 1 wash, (**c**) for 2 washes, (**d**) for 3 washes, and (**e**) for 4 washes. For each wash number condition, spheroid global fluorescent intensity was quantified using Gen5 software (BioTek^®^) (**f**). Otherwise, SUM1315 spheroids were exposed with increasing Olaparib concentrations for 3 h (**g**) or for 6 h (**h**). After the LightSpot^®^-FL-1 staining, pictures were made for each dose for 3 (**g**) and 6 h (**h**) of Olaparib exposure. For each condition, spheroid global LightSpot^®^-FL-1 fluorescent intensity was quantified for 3 h of Olaparib-exposed spheroids (**g**) and for 6 h Olaparib-exposed spheroids (**h**). All pictures were acquired with the Cytation™3 MV (BioTek^®^, M = 4 X, scale bar = 50 µm). Significances between conditions were indicated as ns (not significant; *p* > 0.05), ** *p* < 0.01, *** *p* < 0.001, and **** *p* < 0.0001.

The LightSpot^®^-FL-1 fluorescent probe is able to penetrate into cells and cell masses. Thus, its performance for the detection and quantification of P-gp levels was also analyzed in SUM1315 models. Firstly, to develop a LightSpot^®^-FL-1 staining protocol with this model, SUM1315 spheroids were incubated with 1 µM LightSpot^®^-FL-1 fluorescent probe for 2 h. Then, four washes in D-PBS were carried out to remove the unfixed and unspecific fluorescent compound from the spheroids. At the end of LightSpot^®^-FL-1 incubation, the mean signal fluorescence intensity quantified in spheroids was 49.7 ± 7.7 × 10^3^ FU (Figure 7f). Then, green-fluorescent intensity staining decreased across each washing (Figure 7a–e). Indeed, the mean fluorescent LightSpot^®^-FL-1 intensity significantly decreased to 36.3 ± 10.2 × 10^3^ FU after 1 wash (*p* = 10^−6^, compared to values before washes), to 21.9 ± 11.8 × 10^3^ FU after 2 washes (*p* = 10^−6^, compared to values after 1 wash), to 13.4 ± 7.2 × 10^3^ FU after 3 washes (*p* = 10^−4,^ compared to values after 2 washes) and stabilized to 10.7 ± 2.2 × 10^3^ FU after 4 washes (*p* = 0.1, compared to values after 2 washes). These results suggested that, with these spheroid models, at least 3 washes are required after LightSpot^®^-FL-1 staining to remove the excess and unspecific fluorescent compound.

Secondly, after P-gp staining and methodology development in spheroids with LightSpot^®^-FL-1 tracer, experiments for P-gp level quantification were carried out with this model. As previously carried out on monolayer cultures, SUM1315 spheroids were exposed to increasing Olaparib concentrations, i.e., 10, 50 or 100 µM, for 3 h or 6 h, before being stained with a LightSpot^®^-FL-1 fluorescent probe. In parallel, controls with 0.1% DMSO spheroids were conducted. For all experiments, measured fluorescent intensities were normalized by 0.1% DMSO control spheroid values. After 3 h of Olaparib exposure, no P-gp expression level difference was detected between the 0.1% DMSO control with 100.0 ± 12.7% and the other Olaparib treated spheroids, with 102.5 ± 17.5%, 105.0 ± 17.1% and 111.4 ± 8.2% for 10, 50 and 100 µM, respectively (*p* = 0.95, 0.62 & 0.08, Figure 7g). In contrast, after 6 h of anti-PARP exposure, an increase in P-gp level expression was detected with 133.3 ± 11.6%, 130.7 ± 11.4% and 131.4 ± 14.4% for 10, 50 and 100 µM Olaparib, respectively (*p* = 10^−10^, 10^−10^ & 10^−10^, Figure 7h) than 0.1% DMSO control spheroids with 100.0 ± 12.8%. These results highlighted the performance and sensitivity of the LightSpot^®^-FL-1 fluorescent probe to detect and quantify P-gp overexpression in SUM1315 spheroids, after their exposure to an anti-PARP-targeted therapy.

## 4. Discussion

The development of a tumoral multidrug resistance profile represents a real obstacle in cancer treatment, frequently leading to therapeutic failure [8]. This is a phenomenon in which tumor cells become resistant to multiple structurally and functionally different drugs during treatment [36]. This profile generally appears after initial tumoral exposure to anticancer agents, and mostly requires increasing treatment doses or adapting the treatment strategy [37]. Various molecular mechanisms are involved in multidrug resistance, including membrane efflux triggered by MDR proteins [7]. In particular, the P-gp is the most widely studied member of this family and is associated with tumoral resistance to a wide range of anticancer drugs. Indeed, its expression is often upregulated in tumors, conferring to cancer cells the ability to expulse therapeutic agents and thus limit/evade chemotherapy-mediated cell death. That is why the study of tumoral chemoresistance, established by the P-gp, is a relevant field as a way to optimize cancer treatment and limit therapeutic failure risks [20]. Indeed, the relationship between P-gp expression and treatment efficacy is widely validated for a large variety of cancer types, such as lung, bladder, ovarian, and breast cancers. This is especially of interest for aggressive and challenging tumoral subtypes, such as TNBC. This breast cancer sub-group is commonly associated with a high rate of residual disease after treatment, which increases the risk of relapse in the first 5 years after treatment [38]. One principal cause of therapeutic failure in TNBC treatment is a primary or acquired multidrug resistance profile that can include chemoresistance conferred by the P-gp [39]. Several studies have already demonstrated significant P-gp overexpression after neo-adjuvant chemotherapy that was higher for patients presenting an uncompleted pathological response [40,41]. In addition, it has been highlighted that the upregulation of P-gp expression after the neo-adjuvant chemotherapy of breast-cancer patients was associated with the metastatic spread of lymph nodes [42]. Moreover, other studies had already described the P-gp as a predictive factor of treatment outcomes [21,22]. However, this protein is very rarely used in clinics as a predictive biomarker for the prognosis of the pathology, primarily because of the lack of efficient tools allowing the detection of its presence in clinical samples.

Nowadays, two approaches allow us to evaluate the P-gp expression level at the preclinical step. Firstly, the evaluation of the P-gp involvement in xenobiotic efflux mechanisms can be indirectly studied via competitive fluorescent P-gp substrates, such as rhodamine 123, among many others [43,44]. For this approach, flow cytometry is mainly used, expressly requiring living cells in suspension. Secondly, direct P-gp quantification can be made with enzyme-linked immunosorbent assays, specific antibody immunostaining using Western blotting, immunocytochemistry or immunohistochemistry methodologies [5,45]. These approaches can be applied to cells in suspension, in monolayers, or on tissue sections, and allowed us to specifically detect and localize the P-gp. However, these approaches present difficulties regarding antibody penetration inside cells and/or cell masses and imply expensive and time-consuming experimentation. Thus, they only target extracellular-membrane P-gp, and not the intracellular part [45]. Indeed, the P-gp located on organelle membranes, such as the endoplasmic reticulum and lysosomes, also plays an important role in cellular drug resistance that must not be underestimated [46,47]. Therefore, all of these methodologies present limited interest for routine clinical applications. For this reason, the simple, direct, reliable, and cost-effective detection and quantification of P-gp in tumor cells, and more particularly in tumoral masses, represents a relevant challenge for optimizing treatment efficacy and patient care while limiting the risk of therapeutic failure.

Fluorescence microscopy, based on molecularly targeted imaging agents, may have the potential to overcome such a challenge. Indeed, it provides a way to assess protein location and expression level in biological samples, with subcellular resolution and good sensitivity. As a consequence, over the last decade, there has been a remarkable growth in the development and use of small-molecule fluorescent probes in the biological sciences, particularly in oncology for cancer detection, staging and characterization. In particular, it is well documented that this approach could complement biopsy-based histopathological diagnosis [48]. Small-molecule fluorescent probes typically consist of a target-specific ligand, conjugated through a linker to a small organic fluorophore, for detection in the biological sample. Thus, considering the construction of a P-gp-targeted fluorescent probe, we designed LightSpot^®^-FL-1, a peptidic P-gp inhibitor derivative conjugated to the cell-permeant, bright, and photostable BODIPY-FL fluorophore (Figure 1). This peptidic moiety derives from a series of peptidomimetic P-gp ligands, previously identified as potent and selective non-competitive inhibitors, which reversibly bind to the intracellular domain of the P-gp [49]. From there, we reasoned that such ligands were good candidates to yield an efficient P-gp fluorescent probe after conjugation with a fluorophore.

LightSpot^®^-FL-1 was designed to penetrate inside cells and/or cell masses to specifically label P-gp, thus allowing its direct localization and quantification. Different TNBC cell models were used to evaluate LightSpot^®^-FL-1 as an efficient P-gp probe: DU4475 in a suspension cell line, SW527, HCC1937, MDA-MB-231 and SUM1315 monolayer cell lines, and SUM1315 spheroids.

Firstly, the ability of LightSpot^®^-FL-1 to penetrate and subsist inside cells was checked by flow cytometry analysis, using TNBC SUM1315 and DU4475 cells. Of particular interest, within the first 15 min after cell exposure to the fluorescent probe, a cellular fluorescence intensity 40-fold increase for DU4475 and 5000-fold increase for SUM1315 cells was observed, followed by a stabilization of the fluorescent signal in both cell lines (Figure 2). This suggests rapid LightSpot^®^-FL-1 uptake and binding to its P-gp target. Then, the cell-washing step led to the successful removal of the non-specifically bound LightSpot^®^-FL-1 part. Indeed, after 5 min of washing, a clear decrease of cell fluorescence intensity of 30.9% for DU4475, and of 50.0% for SUM1315 cells, was detected. However, part of the fluorescent LightSpot^®^-FL-1 probe was clearly retained in cells, even after two hours of washing. This part corresponds to 33.3% and 13.6% of the fluorescent tracer signal measured before the washing step, for DU4475 and SUM1315 cells, respectively (Figure 2). These results indicated that the Light-Spot^®^-FL-1 fluorescent probe quickly penetrates and specifically persists in cells.

Thereafter, the P-gp’s selective localization, detection, and quantification by LightSpot^®^-FL-1 staining were demonstrated by fluorescence microscopy. For this demonstration, both TNBC cell models DU4475 and SUM1315 (living or fixed) were exposed to 1 µM of the fluorescent probe for one hour, before a D-PBS wash for another hour, in order to expel excess markers out of the cells. This staining, applied to both living and fixed TNBC cells, highlighted the same membrane and cytoplasmic distribution of the LightSpot^®^-FL-1 compound, with an absence of cell nucleus accumulation (Figure 3). In addition, these results showed 99.5% and 94.4% fluorescent staining intensity similarities between living and fixed cell conditions, for DU4475 and SUM1315 cells, respectively (Figure 3). This demonstrated that LightSpot^®^-FL-1 staining can be carried out on both fresh or fixed cells, giving the same signal distribution and intensity. This property may turn out to be of particular interest for the staining of precious clinical samples. The following experiments were carried out on fixed TNBC cell models.

In order to validate P-gp-sensitive detection and quantification with the LightSpot^®^-FL-1 probe, the FL-1 marking was compared to anti-P-gp immunofluorescent staining using anti-P-gp F4 clone antibodies. These experiments were firstly conducted on DU4475 and SUM1315 cells and highlighted a similar staining signal and cellular distribution on both TNBC models (Figure 4). Strong colocalization between the two sets of labeling was also obtained on DU4475 cells, with a Pearson’s coefficient of 92.1% and a Mander’s overlap coefficient of 100.0%, which highlighted a strong spatial correlation between the two signals. Then, signal fluorescent intensities of both labeling sets were also compared for other TNBC cell lines. The correlation between the two signals for the five TNBC cell lines presented a coefficient determination R^2^ of 0.99 (Figure 5). All these results demonstrated strong similarities between LightSpot^®^-FL-1 staining and anti-P-gp immunostaining signal localizations and intensities. These experiments allowed us to stratify several TNBC cell lines according to their P-gp level of expression, and they are consistent with the literature [35]. Thus, this innovative fluorescent probe transpires to be very efficient for the localization and quantification of P-gp on TNBC monolayer cell cultures.

In addition, the ability of the LightSpot^®^-FL-1 fluorescent probe to quantify the P-gp induction of expression was also checked. Indeed, our previous works had already highlighted the induction of P-gp expression in the SUM1315 cell line after exposure to 50 µM of Olaparib PARP inhibitor, using the Western blotting methodology [28]. Therefore, for these experiments, the TNBC SUM1315 cell line, cultured in monolayer, was also used as a model after their exposure to increasing Olaparib doses (10, 50 and 100 µM) over 3 h. As previously, the LightSpot^®^-Fl-1 staining was achieved alongside anti-P-gp immunostaining. Firstly, a non-significant increase in P-gp expression level was noted after 10 µM of Olaparib exposure, with a 2.8% and 5.7% increase for immunostaining and LightSpot^®^-FL-1 staining, respectively, in comparison to the DMSO control (Figure 6). In contrast, in the presence of 50 and 100 µM of Olaparib, a significant increase in P-gp level was measured with the two methodologies. Indeed, regarding the immunostaining, this increase was 11.4% for both 50 and 100 µM Olaparib concentrations, compared to the DMSO control. In contrast, for the LightSpot^®^-FL-1 staining, the P-gp level increased more strongly up to 55.1% after high-concentration Olaparib exposure, compared to 0.1% in the DMSO control (Figure 6). These results suggest that the LightSpot^®^-FL-1 seems to be more sensitive in the detection of the P-gp overexpression level, in comparison to the immunostaining method. This increased sensitivity permitted by the cell-permeant LightSpot^®^-FL-1 tracer might probably be due to the quantification of P-gp expressed on intracellular organelles, such as lysosomes [50].

This innovative LightSpot^®^-FL-1 tracer presents the ability to penetrate a cell mass. Thus, its performance for P-gp detection and quantification in cell masses was analyzed using SUM1315 spheroid models. Firstly, the P-gp staining method for SUM1315 spheroids was developed. Indeed, after 90 min of SUM1315 spheroid incubation with a 1 µM LightSpot^®^-FL-1 probe, homogeneous distribution of an intense green signal was detected on all spheroid areas. In order to remove unspecific tracer accumulation, spheroids were then severally washed allowing a clear drop of spheroid green-fluorescent signal of 27.0% after one wash, 66.1% after two washes, and 73.0% after 3 washes (Figure 7). Interestingly, upon this step, a stable signal persisted inside the spheroid models, with a similarity of 80.0% (no significant difference observed), despite an additional fourth washing step. Thus, these data allowed us to determine optimal conditions to directly detect and quantify the P-gp expression level inside SUM1315 spheroid models. As previously seen on monolayer cell cultures, the LightSpot^®^-FL-1 probe’s ability to detect changes in P-gp expression level after drug exposure was assessed on SUM1315 spheroids. In these conditions, no significant increase in P-gp expression level was detected after a 3 h short-term Olaparib exposure, with 2.4%, 4.8, and 10.2% of P-gp expression variation for 10, 50, and 100 µM Olaparib doses, respectively, in comparison to DMSO controls (Figure 7). However, after a longer Olaparib exposure of 6 h, the LightSpot^®^-FL-1 compound allowed us to detect a significant P-gp expression level increase of 33.3%, 30.7%, and 30.7% for 10, 50, and 100 µM Olaparib, respectively, in comparison to DMSO controls (Figure 7). These results highlighted the added value of the LightSpot^®^-FL-1 tracer to detect and quantify drug-induced overexpression of P-gp in fixed cell masses such as spheroid models.

The present works allowed the development of the new LightSpot^®^-FL-1 fluorescent probe for the specific localization and quantification of the P-gp chemoresistance protein, inside in vitro preclinical TNBC models. In particular, this innovative tool turned out to be very effective for directly detecting and measuring the basal or induced expression level of the P-gp on both monolayer cultures and spheroid models. One of the added values of the LightSpot^®^-FL-1 is its ability to specifically detect the P-gp on fresh and also fixed cell models. This ability would be particularly interesting regarding precious biological samples and would allow the elimination of P-gp expression level modifications during experiments. Moreover, we demonstrated that this new approach is more sensitive for the detection of P-gp expression level changes than classic immunofluorescence methods. This performance may probably be due to the ability of the LightSpot^®^-FL-1 probe to detect not only the plasma membrane P-gp but also the intracellular P-gp expressed at the membrane organelles [46,47]. Indeed, the P-gp is expressed not only at the plasmid membrane level, but, according to the literature, it is also transiently present on intracellular organelle membranes, such as endoplasmic reticulum, Golgi apparatus and endosome/lysosome vesicles [46]. In fact, all these organelles are involved during the synthesis and traffic of the P-gp. However, the P-gp is maintained on the lysosome membrane, playing an important role in tumoral chemoresistance via lysosomal drug sequestration and degradation [50,51]. Therefore, this approach would be more complete for the detection of the global P-gp cell expression level. For this, other works are currently in progress to characterize more precisely the intracellular LightSpot^®^-FL-1 staining. All these characteristics make the LightSpot^®^-FL-1 probe very effective for the quantification and localization of the P-gp inside multicellular biological structures. In particular, this innovative tool is very promising for improving studies of the P-gp and for the clinical diagnosis of tumoral chemoresistance established by this protein. More specifically, the LightSpot^®^-FL-1 may help to re-introduce the tumoral P-gp expression level as a predictive biomarker of therapeutic failure or cancer relapse. This new clinical approach may turn out to be very useful as a way to improve tumor treatment that is all the most important in challenging cancer subtypes such as TNBC. In this field, our current works are focused on the development of the LightSpot^®^-FL-1 probe on clinical samples for the evaluation of tumoral chemoresistance.

## 5. Patents

These present works follow the international patent filing # PCT/EP2020/077694 from 2 October 2020, by UCA/INSERM/BIOMARQUEURS [34].

## Data Availability

The data presented in this study are not publicly available and are available on request from the corresponding author.
